# Improvement in the pre-hospital care of recreational drug users through the development of club specific ambulance referral guidelines

**DOI:** 10.1186/1747-597X-3-14

**Published:** 2008-06-06

**Authors:** David M Wood, Shaun L Greene, Graham Alldus, Denise Huggett, Michelle Nicolaou, Kerry Chapman, Fionna Moore, Kim Heather, Nicola Drake, Paul I Dargan

**Affiliations:** 1 Guy's and St. Thomas' Poisons Unit, Guy's and St Thomas' NHS Foundation Trust, Avonley Road, London, SE14 5ER, UK; 2 Lambeth Police LGBT Liaison Team, Brixton Police Station, 367 Brixton Road, London, SW9 7DD, UK; 3 Emergency Department, Guy's and St Thomas' NHS Foundation Trust, Westminster Bridge Road, London, SE1 7EH, UK; 4 Vauxhall Gay Business Forum and Fire Nightclub, South Lambeth Road, Vauxhall, London, SW8 1RT, UK; 5 Vauxhall Gay Business Forum and Metropolitan Police Safer Neighbourhoods, Prince's Ward, Vauxhall, London, SW8, UK; 6 London Ambulance Service NHS Trust, 220 Waterloo Road, London, SE1 8SD, UK

## Abstract

**Background:**

Previously developed 'club guidelines' developed for club owners and promoters have tended to focus more on the legislative aspects of clubs, rather than the medical management of unwell clubbers within club environments. Despite this lack of guidance on the management of unwell clubbers, a significant proportion of clubs have 'club medic' rooms for managing these individuals. However, due to the lack of specific guidance on the training of staff working in these rooms and guidelines on when an ambulance should be called for an unwell clubber, there have been instances previously where clubbers have been inappropriately managed within the club environment, and often referred to hospital only after significant physiological derangement has occurred, thereby leading to an increased risk of morbidity and mortality.

**Methods:**

We identified owners and promoters of local club venues within the catchment area of our Emergency Department and working jointly with them and other key stakeholders, in particular the London Ambulance Service and Metropolitan Police, identified strategies to improve pre-hospital care for clubbers who become unwell as a result of recreational drug use. These included developing guidelines detailing indications for ambulance transfer to hospital for clubbers with recreational drug toxicity and the training of club medic staff to use the guidelines

**Results:**

Following the initial development of a pilot set of guidelines, an audit of their use identified training needed relating to the assessment of unwell clubbers with recreational drug toxicity and revisions required to the pilot version of the guidelines. After training related to the revised guidelines, all the club medic staff were confident in their ability to assess unwell clubbers with recreational drug toxicity, the use of the guidelines and also when to call an ambulance.

**Conclusion:**

Working with key stakeholders in the local community, we have developed guidelines that can be used to improve the pre-hospital care of clubber unwell with recreational drug toxicity, and demonstrated that individuals with a variety of medical knowledge can be trained to use these guidelines. Wider dissemination of these guidelines, both regionally, nationally and potentially internationally, may help to reduce the pre-hospital morbidity and mortality associated with recreational drug toxicity encountered in club environments.

## Background

The use of recreational drugs is common throughout the world, and in particular in the UK [1]. Previous studies have shown that a significant proportion of individuals entering treatment programmes with problematic use of recreational drugs, such as MDMA, gamma-hydroxybutyrate and ketamine, have had previous attendances in Emergency Rooms [2]. It is not clear from these studies whether ED presentations were directly from a nightclub, or from some where else (e.g. home).

Within the UK, the "Safer Clubbing" guidelines have been developed for nightclub owners and promoters; these focused more on the legislative and security aspects of clubs, rather than the medical management of unwell clubbers within club environments, particularly those with recreational drug toxicity [3]. Some authors have discussed the provision of 'first aid' facilities within club venues, although this has not been specifically for problems relating to recreational drugs [4]. Despite the lack of guidance concerning the management of individuals with toxicity following recreational drug use, a significant proportion of clubs now have dedicated 'club medic' rooms where individuals with recreational drug toxicity can be assessed and managed. These rooms have a range of medical equipment available for the assessment of individuals, and the experience of club medic room staff ranges from basic first aid training to paramedic/nurse-based training [5].

Our experience has been that club owners/promoters maybe reluctant to call an ambulance for clubbers with recreational drug toxicity, because of concerns that this could affect their licence. This has led to clubbers being inappropriately managed within the club environment, and potentially being referred to hospital only after significant physiological derangement has occurred, thereby leading to an increased risk of morbidity and mortality. This led us to work with key stakeholders in the pre-hospital setting to try and develop strategies to improve pre-hospital care for clubbers who become unwell as a result of recreational drug use. This included developing guidelines detailing indications for ambulance transfer to hospital for clubbers with recreational drug toxicity and the training of club medic staff to use the guidelines.

## Methods

### Identification of key stakeholders and issues relating to recreational drug toxicity

Following our experience of individuals being referred late from club venues with recreational drug toxicity, we identified owners and promoters of local club venues within the catchment area of our ED, a process aided by the local police service. Daytime visits were undertaken by DW, KH and GA to assess the various club medic rooms, facilities available, and the background and previous training of staff working within these rooms; these visits involved a general inspection of the facilities and discussion with relevant club staff, which was felt to be best done during the daytime when the clubs were not open and they had more time available.

### Development and implementation of guidelines

**Figure 1:**
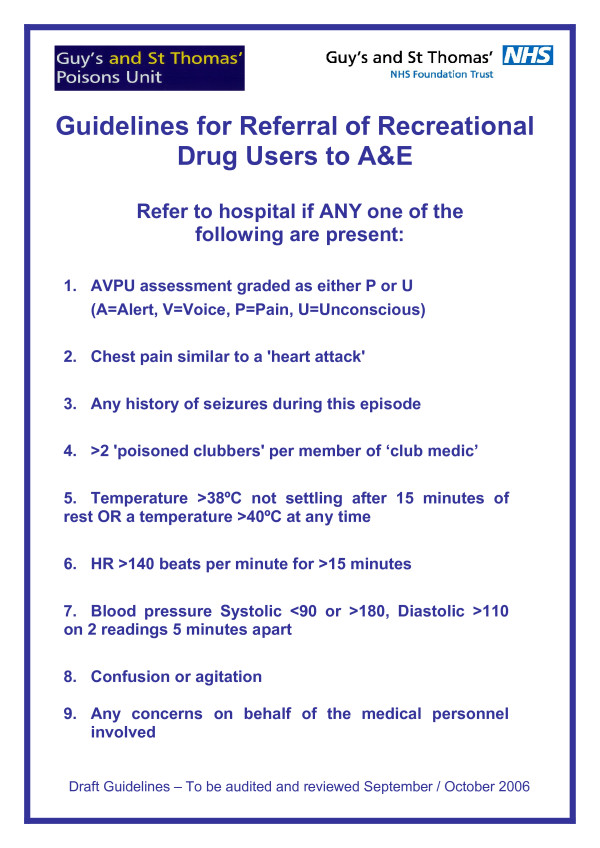
Initial referral guidelines developed on when to refer an unwell clubber with recreational drug toxicity to hospital.

Using the above data and a literature search to identify any previously published data on pre-hospital assessment of individuals with recreational drug toxicity in a nightclub environment, we developed guidelines to enable club medics to identify clubbers with physiological evidence of recreational drug related toxicity requiring or likely to require ED assessment and treatment using basic physiological parameters, enabling club medics with minimal medical knowledge to accurately assess individuals (Figure 1). Prior to implementing the guidelines we ran a training session for all club medics to introduce the guidelines and discuss general issues in the management of patients with recreational drug toxicity, using case scenarios.

### Audit and review of initial guidelines

**Figure 2:**
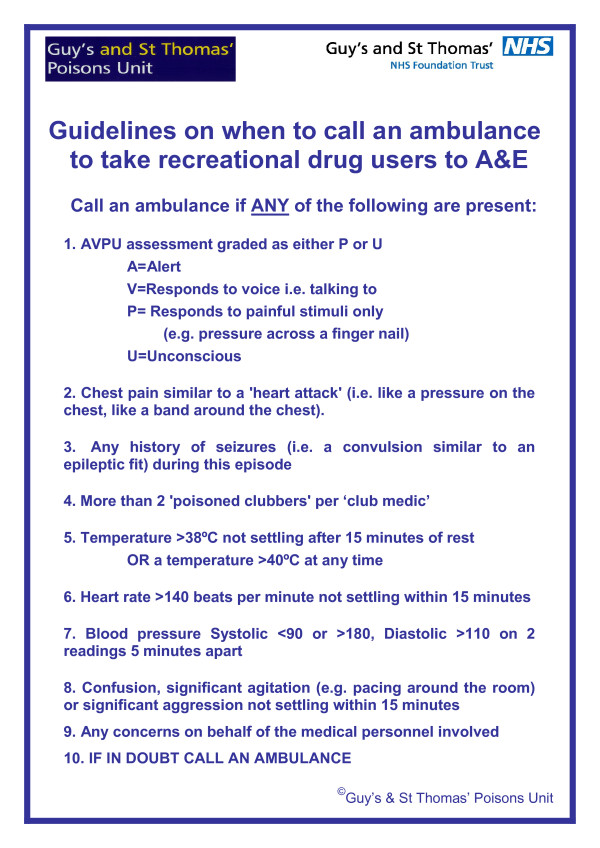
Revised finalised version of the guidelines on when to call an ambulance for a clubber with recreational drug toxicity, developed following the audit of the original version of the guidelines.

After introduction of the initial referral guidelines, management of all individuals attending a club medic room with recreational drug related toxicity was assessed using a standardised proforma, completed by medic room staff over an 18 day period in November 2006. Presenting clinical parameters (heart rate, blood pressure, conscious level as assessed using the AVPU score and temperature), fulfilment of defined transfer criteria and final disposition were recorded. These forms were returned directly by individual clubs using anonymous pre-paid envelopes addressed to the principal investigator (DW). Each of the audit forms was assessed by DW/PD/DH and a consensus opinion on the appropriateness of disposition and management was reached for each case and patterns of deficiencies were recorded. This was used to undertake a review of the initial guidelines and produce a revised guideline taking into account any issues identified in this audit (Figure 2).

### Implementation of revised guidelines and review of ability to use the updated guideline

These revised guidelines were distributed to all clubs involved in the study and we ran a half day training session for club medics from all 8 clubs involved in this study on the revised guidelines. This was facilitated by GA and run by DW/PD/KH using similar case scenarios to the pre-implementation training session. The results of the audit were discussed with the club medics and they were given the opportunity to provide further feedback on the guidelines. At the end of this training session club medics were asked to complete a structured questionnaire indicating whether they felt competent in: i) assessing unwell clubbers following recreational drug use; and ii) using the revised referral guidelines and whether further training was required to manage individuals presenting with recreational drug related toxicity.

## Results

### Identification of key stakeholders and issues relating to recreational drug toxicity

Club owners and/or promoters in all 8 club venues in the concentrated area within the catchment area of our large inner city ED were recruited to take part in this pilot study. These venues largely cater to the MSM (men who have sex with men) community, although not exclusively. All of the venues had a club medic room facility, although the size, facilities and ventilation/air conditioning within the rooms varied greatly. Additionally the training of the club medics varied from venue to venue, although in general they were supplied by one or two agencies and/or employed directly by the club venue. Given the variety of facilities and training of staff, it was decided that the guidelines described here would be written to cover all levels of medical training and also be implementable with the minimum of equipment required. The recommended equipment inventory was a bed, thermometer, sphygmomanometer, watch/clock with second hand, cold water and that the facility should be housed in a quiet environment with adequate ventilation.

We have identified that the most common agent involved recreational drug toxicity presentations to our ED from the club venues is GHB and its analogues, and that the majority of individuals do not co-ingest ethanol [6]. Additionally MDMA toxicity is also commonly seen in this population, but that methamphetamine, whilst having high media attention concerning use in this clubbing population, is not a significant issue [7].

In terms of additional key issues, relating to delay in transfer of clubbers who had become unwell following use of recreational drugs, these fell broadly into 2 categories. Firstly, those individuals with GHB or analogue toxicity with significant depression of CNS and/or respiratory function; secondly, those individuals with sympathomimetic toxicity with hyper-pyrexia and/or cardiovascular toxicity (tachycardia, hypertension). The guidelines described here have been designed to ensure that individuals with significant toxicity in these categories are easily identified and an ambulance can be called.

### Development and implementation of guidelines

Since there are no published guidelines on pre-hospital assessment of clubbers with recreational drug toxicity we decided to develop a set of guidelines using the multidisciplinary expertise of the study group. An initial set of referral guidelines for the unwell clubber with recreational drug toxicity were drafted jointly by the Clinical Toxicology team at Guy's and St Thomas' Poisons Unit (DW/PD/KH/SG) and the Emergency Department (ED) at St Thomas' Hospital (ND/DH and colleagues). These draft guidelines were circulated to other members of the multidisciplinary team including club staff and managers from local ambulance stations for comments. These guidelines aimed to enable club medics to identify clubbers with physiological evidence of recreational drug related toxicity requiring or likely to require ED assessment and treatment, and provided definitive indications for ED transfer. Guidelines utilised basic physiological parameters, enabling club medics with minimal medical knowledge to accurately assess individuals. The initial version is shown in Figure 1. Prior to implementing the guidelines we ran a half day training session for all club medics to introduce the guidelines and discuss general issues in the management of patients with recreational drug toxicity. This was facilitated by GA and run by DW/PD using case scenarios to illustrate how to utilise the guidelines and discuss general issues relating to recreational drug toxicity.

### Audit and review of initial guidelines

#### Completion of audit forms

**Figure 3:**
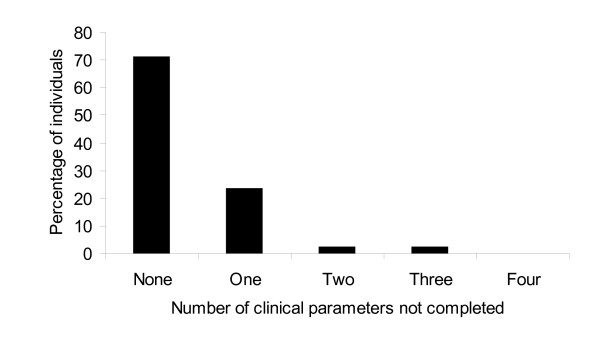
Percentage of initial clinical parameters, part of the decision process for calling an ambulance, that were missing following assessment of an unwell clubber following use of recreational drugs.

Forms were returned for 42 individuals who attended a club medic room between 11^th ^November 2006 and 27^th ^November 2006. 12 (28.6%) of forms had at least one clinical observation missing from the initial assessment of an unwell clubber presenting to a club medic room (Figure 3). The most common clinical parameter not recorded in these forms was temperature [n = 8; 66.7%]; followed by heart rate [n = 3; 25%], blood pressure [n = 2; 16.7%] and AVPU score conscious level [n = 2; 16.7%].

#### Final disposition of clubbers

Of those clubbers who presented to a club medic room, 19 (45.2%) were allowed home after review, an ambulance was called for 7 (25%) and all were taken to hospital; data was not complete for the remaining 16 patients, although the forms record that no ambulances were called for this group of clubbers. In all cases where an ambulance was called for a clubber with recreational drug toxicity, it was deemed appropriate as the individual had met one or more of the criteria specified in the guidelines for calling an ambulance.

#### Appropriateness of management

The major problems identified following the introduction of the initial referral guidelines related to the non-referral to hospital of those individuals with an AVPU score of P or U (11, 23.9%), those having had a 'seizure' following use of recreational drugs (5, 10.9%) or those who were agitated or aggressive in the club medic room (7, 15.2%). Where the clinical parameters had been correctly completed on the form (n = 30; 71.4%), the management and final disposition of the patient on review of the form was deemed to be appropriate in 22 (73.3%) of cases. For the 8 remaining cases, management was deemed inappropriate due to non-referral to hospital for seizures (n = 2; 25%), an AVPU score of P or U (n = 5; 62.5%) or both seizures and an AVPU score of P or U (n = 1; 12.5%)

### Implementation of revised guidelines and review of ability to use the updated guideline

All 16 club medic room staff who attended the training session run at the time of introduction of the updated guideline completed a questionnaire at the end of the session. 14 (87.5%) felt confident in assessing unwell clubbers following use of recreational drugs; the remaining 2 (12.5%) felt 'generally confident'. 100% of individuals felt confident in using the referral guidelines in deciding when a clubber with recreational drug toxicity should be referred to ED/an ambulance called. No individuals surveyed felt that additional training was required following this 'one off' training session.

## Discussion

Recreational drugs are commonly used in the UK, particularly within the nightclub environment [1,8,9]. Recreational drug toxicity is a common reason for presentation to the ED [6,7,10]. Despite this there are no published guidelines on the assessment and management of individuals with recreational drug toxicity in the pre-hospital and particularly the nightclub environment.

We have shown that multidisciplinary team working together with both medical and non-medical individuals and organisations involved in the pre- and in-hospital care of unwell recreational drug users can be used to develop strategies to improve pre-hospital management and identification of individuals that require immediate hospital assessment. After appropriate training, club medics felt confident using the guidelines to assess individuals with recreational drug toxicity in the pre-hospital environment. In addition, there has not been a significant increase in the number of recreational drug related presentations to our clinical toxicology service suggesting that the guidelines have not resulted in inappropriate referral to hospital. When introducing guidelines it is good practice to review how easy they are to complete and to ensure that they are easy to follow. It is also important that guidelines are not introduced without appropriate training in their use; and after introduction that the appropriateness of this training is assessed to identify shortfalls to allow the guidelines to be adapted and additional training provided, as we have done in this study [11]. We have not specified the minimum training requirements for club medics, since the guidelines were designed so that individuals with qualifications such as 'First Aid at Work' would be able to use them. This is important, since with wider implementation to nightclub and night-time venues that do not have designated club medic staff, we would hope that these guidelines could still be implemented and used by other appropriately trained members of staff.

The main limitation of our study is that because of patient confidentiality, particularly in the pre-hospital setting, we have not been able to assess whether they have had a direct impact on the speed in which an ambulance is called for an unwell clubber with recreational drug toxicity and their transfer to our ED and/or whether the guidelines have resulted in inappropriate referrals to hospital. The use of anonymous pre-paid envelopes, ensured that the information was not shared directly with the police, and therefore increased the likelihood that the clubs would be involved, however the disadvantage of this is that it meant we were unable to identify individual patients and link the data from the club with their hospital attendance, where appropriate. Finally, in this study we have not tried to correlate the clinical condition of the patient with either self-reported use of recreational drug(s) or toxicological screening of blood and/or urine samples. These guidelines were developed to identify individuals with significant toxicity following use of recreational drugs, irrespective of the actual drug(s) ingested. We are currently investigating the feasibility of a pre-hospital study looking at self-reported use of recreational drugs compared to findings on toxicological screening of these individuals.

In the UK there are no specific guidelines used within club environments to allow staff to easily assess unwell clubbers with recreational drug toxicity. The original "Safer Clubbing" document introduced in 2002 had guidelines for club owners/promoters and legislative authorities including club licensees, although the focus of this document was on the physical environment of clubs, security and legislative issues related to recreational drugs [3]. In particular, there was limited content related to the clinical assessment and management of recreational drug toxicity in the pre-hospital club setting. We have been working with the London Drug Policy Forum and other interested parties (drug and alcohol teams, Department of Health, Home Office, HIV/men who have sex with men outreach workers, Terence Higgins Trust, Licensing Authorities) in updating the "Safer Nightlife" document to include additional information relating to clinical aspects of recreational drug toxicity, incorporation of our ambulance referral guidelines, and the need for training related to the pre-hospital management of recreation drug toxicity. Previous published studies have shown that users of recreational drugs continue to use whilst travelling abroad on holiday, and may in fact increase their use [12]. Therefore the development of these guidelines are not necessarily specific to the location and/or country in which they have been developed, since the same types of toxicity are likely to be seen in other areas/countries. We hope that the publication of the updated "Safer Nightlife" document, the wider training of club medics in the management of individuals with recreational drug toxicity and the use of these ambulance referral guidelines will lead to an improvement in pre-hospital management of recreational drug toxicity on a wider scale outside of the pilot project area. During implementation of the guidelines in other geographical locations, there maybe need to consider developing a generic training package to accompany the guidelines, and their implementation elsewhere should be assessed appropriately.

## Conclusion

We have successfully worked with a multidisciplinary team to develop guidelines and training for club medic staff on the assessment of individuals with recreational drug toxicity in the club environment and in particular when an ambulance should be called. These guidelines have been well received by the club medics. We believe that wider implementation of these guidelines, along with training in their use, will help to reduce pre-hospital morbidity and mortality from toxicity following use of recreational drugs in club environments.

## Competing interests

DW and PD have acted as scientific advisers to the UK Advisory Council on Misuse of Drugs (ACMD) and the European Monitoring Centre for Drugs and Drugs Addiction (EMCDDA). DW and PD are members of the steering group involved in the rewriting of the "Safer Nightlife" document.

## Authors' contributions

The initial project was idea came from SG, and was led by DW with the assistance of all the other parties. The pilot guidelines were developed by DW and PD and then revised by ND, SG, KH and DH with input from other clinical colleagues at St Thomas' Hospital, before implementation facilitated by GA, KC and MN and training associated with the guidelines undertaken by DW, PD, KH and DH. The audit of the pilot guidelines was lead by DW with the assistance of GA, KC and MN and the analysis was undertaken by DW, PD and DH. The first draft of this paper was written by DW and PD and all authors have reviewed, revised and contributed to the finalised draft.
